# Essay on Pyrexia, or Symptomatic Fever

**Published:** 1838-01

**Authors:** 


### Art. XV.

*An Essay on Pyrexia, or Symptomatic Fever, as illustrative of the Nature of Fever in general.* By Henry Clutterbuck, m.d.—*London*, 1837. Pp. 136.

This essay, the production of one of the most eminent of the physicians of London, has disappointed us. The vagueness of the title-page pervades most of the subsequent pages, and we close the book with a very indistinct idea of the author's precise intention in publishing it.

Several years ago, (about thirty,) and before the birth of the theory of Broussais, according to which every fever is symptomatic, and symptomatic of inflammation of the mucous membrane of the stomach and intestines, Dr. Clutterbuck published his views on the same subject; first in an inaugural thesis at Glasgow, and subsequently in an enlarged form, in an "Inquiry," which appeared three years afterward. According to these views, which are also maintained in the present essay, every fever is really symptomatic, although, with the same peculiar employment of words which leads to the use of pyrexia as the synonym of symptomatic fever, Dr. Clutterbuck calls such fevers as are, according to his own theory, symptomatic of cerebral inflammation, *strictly idiopathic.* The terms, thus, as we conceive, misapplied, are employed in almost every page. Separating the fevers symptomatic of all other inflammations from what is called, and what the author calls, idiopathic fever, Dr. Clutterbuck ascribes the latter, not to inflammation of the gastro-intestinal mucous membrane, but to inflammation of the brain. According to both theories, there is no such thing as a fever to which the term idiopathic can be properly applied, that term being significative of a primary general disorder of the system; of which they do not admit the possibility. Dr. Clutterbuck retains the term, we do not know why: M. Broussais and his followers reject it with ridicule and scorn; looking upon the idea of an essential fever as an abstraction, a mere imagination, a figment of the mind.

The authority of such names as those of Broussais and Clutterbuck might well discourage opposition, if the great theorists were not so much opposed to one another. Their theory would be unassailable, if they were equally fortified by union in all the points of it. To them we have to ascribe the more willing admission made by modern practitioners of the existence of local inflammations in fevers, and the importance of attending to them. But this did not suffice them. Each, much impressed with the frequency as well as importance of these complications of fever, raised unto himself an inflammatory doctrine of fever, to the discountenance of the essentiality of fever itself. Both practical men, both good observers, the theories they built up are such, and rest on such proofs, that the established truth of either would inevitably destroy the other. This is a curious illustration of the difficulty of observation, and the fallacy of experience. That both the theorists are entirely wrong, and that each has through life been in full pursuit of one idea, and the idea of each wholly incorrect, is inconceivable. There must be some points on which each is right,—some neutral ground on which each is safe,—although each may deny the stability of that very territory. We suspect, also, that there is some common ground in which they each and equally wander into error. Where they differ, each is to a certain degree right; where they agree, we apprehend that both are wrong. Such a spectacle must be infinitely diverting to those unskilled in professional niceties; although the high character of the combatants, as well as the seriousness of the question, involved in such disputes, has caused the medical profession to look on the contest with interest and respect.

Dr. Clutterbuck says *his* doctrine has been adopted "by not a few," with little reservation, and he claims for it the high merit of having materially contributed to improve the practice in fever, by dispelling the idea of debility being an essential part of the disease, and teaching that the debility arises from a previous state of vascular excitement "in the brain more especially." This is, indeed, no small merit, and we believe Dr. Clutterbuck to be justly entitled to it. We learn from the publications of the time, that, about the period when his Inquiry was published, wine and bark were indiscriminately given by many practitioners in every case of fever. The first part of the Inquiry, the work in which Dr. Clutterbuck's views were published, contained the general doctrine; the second part, which is to shew the application of the doctrine to the *varieties* of the disease, has not yet appeared. In the mean time we are not left to suppose that Dr. Clutterbuck has modified his original views, the truth of which, he says, has only become the more firmly fixed in his mind by subsequent reflection. The object of the essay now before us is "to endeavour to shew that a febrile state of the system, or what is technically denominated *symptomatic fever*, is always a *secondary* affection, the result of inflammation, and of no other cause." (*Introd.* p. vi.) We need scarcely observe, that any fever, which is properly a symptomatic fever, must be secondary; but the question is, whether or not the primary accident is always inflammation. For the opinion that it is always inflammation, Dr. Clutterbuck claims importance in relation to diagnosis; as it must lead the practitioner carefully to seek in every case for an inflammatory cause. This, it is evident, may be advantageous or not, as the truth of the theory is established or doubtful. A prudent practitioner will be careful not to overlook any existing inflammation; but the practitioner who always asserts that there *must* be inflammation going on somewhere, will scarcely, unless always right in his opinion, be so prudent in his measures as the welfare of the patient requires; although Dr. Clutterbuck does not forget to mention that the pyrexia, which he considers secondary, may materially affect the character of the inflammation, which, according to him, is the primary disease; and although he admits that the pyrexia is *sometimes* the more immediate cause of the fatal termination, and that, even in brain-fever, a secondary inflammation of some other organ may supervene, and constitute the essential part of the disease.

As regards the subject of Dr. Clutterbuck's essay, the *symptomatic* fevers; the very name implies that they are of *secondary* origin. They must be symptomatic of something, or not symptomatic at all. But the main assertion involved is, that the febrile state, whenever secondary, is symptomatic of inflammation alone; and that, when no local inflammation can be anywhere detected, as in fevers in which the sensorium is more or less affected, there is always inflammation present, and the brain is the organ inflamed.

It is impossible not to feel a conviction in the outset, that Dr. Clutterbuck's opinions, as well as those of M. Broussais, who would say just as much of the gastro-intestinal mucous membrane, are opposed by undeniable experience of cases in which neither the symptoms, from the beginning to the decline of the malady, nor the treatment found successful, indicate the presence of a state that can, with any propriety, be termed inflammatory. But no one can suppose such cases to have escaped Dr. Clutterbuck's attention; and this it is which gives a peculiar interest to the enquiry. Restricting the term symptomatic fever to fevers arising from all other inflammations, he considers idiopathic fever (which term he rather inconsistently retains, and often employs,) to be a local disease,—an inflammation of the brain; and he admits no other kinds of fever, excepting intermittent fever, which he looks upon as a distinct and specific disease; thus avoiding, as it appears to us, the inconvenient illustration which it affords, together with fever from the absorption of morbid poisons, of fever without local inflammation of any kind for its cause. Dr. Clutterbuck considers the prostration of strength, the "viribus præsertim artuum imminutis" of Cullen's definition, as not being a part of the pyrexia, or symptomatic fever, but only of the *proper* or *idiopathic* fever; which terms he so often introduces in this manner as to leave us in doubt whether or not he restricts them to inflammation of the brain. He gives a minute description of what he calls pyrexia, or symptomatic fever, without, however, making it very clear what he is really describing. After more than one perusal of the first section of the work, it leaves no distinct impression on the mind. We do not see, in the descriptions it professes to give, pictures of disease, but rather ingenious views, shaded by a one-sided theory. Nor can we wonder at Dr. Clutterbuck's ready imagination of the existence of inflammation of the brain in every case of fever not arising from some obvious inflammation existing elsewhere, when we find him disposed to consider the febrile condition induced by the use of mercury as so induced by a state bordering on inflammation of the internal surfaces of the blood-vessels; and even to look on pregnancy and menstruation, because often attended by febrile symptoms, as conditions having at least a great resemblance to inflammation. All our previous notions of pathology are confounded rather than corrected by the assertion, that "menstruation, in fact, in many females, is a state nothing short of actual uterine inflammation, and calls for an appropriate treatment." In like manner, Dr. Clutterbuck ascribes the febrile condition following a debauch in eating and drinking, the next day's sorrow of the debauchee, to inflammation of the mucous membrane of the œsophagus and stomach. In some of the previous pages, he points out, as diagnostic of what he calls "pyrexia, or symptomatic fever," heat of skin, frequency of pulse, and a furred tongue; and insists on idiopathic fever, or what is in his opinion always a brain-fever, being distinguished by the addition, or at least the presence, of "*pain in the head*, disordered sleep, prostration of muscular strength, and other disturbance in the state of the sensorial functions." The victim of hesternal conviviality suffers from all these; and we do not see why his case is not classed by Dr. Clutterbuck among brain-fevers.

The symptoms, which are decidedly febrile and symptomatic, are, however, considered by him as symptomatic of local inflammation; and he locates the inflammation, not in the brain, but in the œsophagus and stomach. We cannot imagine an opinion standing on a looser foundation. The force of imagination required to conceive the entity of fevers is mere feebleness compared with that which can be satisfied that there is any inflammation at all in such a case. There is a state of irritative fever; symptomatic doubtless, but not of inflammation.

Dr. Clutterbuck lays particular stress on the relief of symptomatic fever, as a means of acting upon the original inflammation, of which, according to him, fever is always symptomatic. He seems to ascribe tubercles in the lungs, in almost every instance, to an inflammatory origin; and he considers the relief of the hectic fever as the most important circumstance in the treatment of that malady. The following extract sufficiently illustrates the views he takes of this subject.

"A febrile state of body argues the existence of inflammation, equally in the weak as in the strong. The intrinsic nature of the disease is the same, and the treatment is governed by the same general principles. It is only necessary that the remedy should be properly adjusted to the existing circumstances; and, with this limitation, bloodletting will be found both as safe and as effectual in the one case as in the other.

"The case of hectic fever, as occurring in pulmonary consumption, serves to illustrate what has now been said. In the confirmed state of this disease, a cure is rarely to be expected from this or any other means. Yet much may be done to mitigate the violence of febrile action, (the hectic;) by doing which, much suffering is spared to the patient, and life often prolonged to a considerable extent. These important objects may be accomplished in many instances by bloodletting, repeated from time to time, but always in small quantities, as from three or four to five or six ounces at a time; the rule being, in such cases, so to draw blood as not sensibly to disturb the functions or feelings of the patient. When used with these precautions, and while a tolerable share of general health still remains, the good effects of small and repeated bleedings are often strikingly displayed, by their relieving all the most distressing feelings of the patient, inducing quiet sleep, and restoring appetite, if lost. And I may further add, that, if the disease admits of a cure, (as undoubtedly is sometimes, though rarely, the case,) it is most likely to be accomplished by such means; and this I have seen in different instances. I consider it, however, to be essential to this mode of treating pulmonary consumption, whether undertaken with a palliative or curative intention, that the patient should be allowed to take food, either animal or vegetable, as his habits and inclinations may lead him. And it is no small recommendation of this practice, that not only is appetite usually excited by such small bleedings, but animal food may then be taken without producing that feverish excitement which it is otherwise apt to do." (P. 97.)

In inflammation of the mucous membranes, although Dr. Clutterbuck observes that they have a tendency to spontaneous subsidence, by an increased secretion of mucus, and although the disease may be prolonged if bloodletting is carried so far as to weaken the system considerably, he does not admit that there is anything so depressing in the nature of the inflammation as to preclude the use of that remedy altogether; but, on the contrary, when the symptoms are severe, considers bloodletting as useful, and even as necessary, in the treatment of catarrh, diarrhœa, and other inflammations of the mucous membrane, if recent, as in inflammation of any other structure.

At the outset of ordinary idiopathic fever, which we are to recollect is, according to Dr. Clutterbuck's views, brain-fever, or fever dependent on inflammation of the brain, he deems the administration of emetics, several times repeated, at intervals of a few hours, highly useful, on the principle of counter-irritation. He questions the good effects of large doses of tartarised antimony; but places considerable reliance on digitalis, which, by its specific power of lessening the frequency of the pulse, and thereby diminishing animal heat, he thinks particularly calculated to subdue febrile action; particularly in the hectic fever of consumption, in which, he says, "it commonly diminishes, and sometimes subdues altogether, the febrile symptoms. Under its beneficial operation, the night-sweats disappear, the sleep becomes calm and refreshing, and the appetite is restored. The progress of the disease towards its usual fatal termination is often arrested for a time; and, more than this, a cure is sometimes thus effected, especially when the circumstances admit of its being used in combination with small and repeated *bleedings*, as already mentioned." (P. 105.)

Dr. Clutterbuck is of opinion that opium, in the generality of cases, rather aggravates than diminishes febrile action, whilst it also checks the secretions and masks the symptoms. Of nitre, the acetate of ammonia, and the citrate of potass, he speaks slightingly; more so than, we think, these convenient and often-used remedies deserve. He lays just weight on the regulation of the food, drink, air, and exercise of the patient in all cases of symptomatic fever; observes that many chronic inflammations, when accompanied with a febrile state of body, will yield to persevering abstinence, where a more active treatment has been useless. Pulmonary consumption has, he thinks, given way, in many instances, to the continued use of a milk and vegetable diet; and he thinks he has seen cancer arrested in its course, or its symptoms much mitigated, by the adoption of Dr. Lambe's system of vegetable diet.

Dr. Clutterbuck devotes several pages to endeavouring to shew that the able authors of the Elements of Medicine, Drs. Bright and Addison, differ very little from him respecting the nature of the fevers usually called idiopathic. To us, those respectable authors appear to concede no more to Dr. Clutterbuck than the majority of British pathologists would cheerfully do. They would doubtless acknowledge, as we do, the respect due to any opinions entertained by a physician so experienced, and of so high a reputation as a judicious practitioner. They would probably abjure the notion of mere debility in the commencement of fever; and would search for and recognize the presence of local inflammation whenever it existed, and, in consequence of its presence, or without it, in consequence of the presence of great vascular excitement alone, would promptly bleed the patient. But it is equally evident that they think the inflammation, when present, a secondary phenomenon in the order of the occurrence, preceded by several febrile phenomena; and that they believe these phenomena, even when indicating disturbance of the cerebral functions, may be associated with a morbid condition of the brain which is yet not inflammation. It would seem little more than a question of terms, if the head-symptoms always kept pace with the increasing severity of the febrile symptoms; or if the increase so often observable after the fever has continued for ten days were not frequently found to keep pace with, perhaps to depend upon, the development of inflammation in some other organ, without sufficient evidence, or any evidence, of the brain itself being also inflamed. That there are many cases of fever, also, which run through their whole course with sufficient evidence of a morbid condition of many functions, not only of the sensorium, but of other parts of the system, in which it is no more reasonable to assume the existence of inflammation of the brain than of other organs, the uninflamed state of which is more easily verified, is, we suppose, unquestionable; and that cases of fever may be fatal either without exhibiting any signs of inflammation on examination after death, or such marks of it as are inadequate to account for death taking place, equally so. Our own observation is entirely baffled, if there are not many cases of simple fever, properly so called, cases of general disturbance without a primary inflammatory cause, in the course of which, and in the space of several weeks, irritations of the intestines, of the brain, and of the lungs successively appear, without inflammation, and without the invariable appearance of cerebral irritation as the first link in the chain, however early it may appear. When inflammation is decidedly present in fever, too, the progress and character of the fever are not, we presume, always or often found to be those of a simple symptomatic fever, wherever the inflammation may be; but are apparently influenced by some unknown causes, productive, in different epidemics, of more or less depression of the vital powers, and corresponding inability to bear lowering measures. All these are points which have been ably demonstrated by many careful pathologists; and such as we should have expected to find weight with more exclusive theorists. Although we are inclined to believe that practical benefits have arisen from the views taken by Dr. Clutterbuck, and those entertained by M. Broussais, (we particularly allude to the greater attention drawn towards inflammations occurring in fever,) we should much question the general soundness and safety of any practical measures whatever, *generally* appiled to fever, and without especial regard to many or all of the above circumstances. The adaptation of the remedial means to the different cases met with in practice, according to the preponderance of the local or general symptoms, is one of the most delicate points which the physician is called upon to determine; and doubtless he will be most likely to determine accurately whose theory does not indispose him to scrutinize the nice evidence of the combination before him.

During the thirty years which have intervened between the publication of Dr. Clutterbuck's Inquiry and the present Essay, ample evidence has been collected, in all countries, of the variable types of fevers, to which Sydenham, long before, directed especial attention. They were insisted upon, with much force, by Dr. Wilson Philip as soon as the enquiry appeared, and few practitioners are now wholly inobservant of them. We cannot suspect Dr. Clutterbuck of overlooking them; but we think that both his theory and the practice it inculcates are somewhat dangerously opposed to the caution required in the treatment of fevers. In private practice, the want of such caution passes almost unobserved; but in public institutions the error has been shown in its worst consequences again and again. If the older practitioners erred with wine and bark, the moderns have not always been free from error with the lancet and leeches; and in both cases the hurtful practice arose from erroneous theories, from conclusions which became false when they became general. It may amuse as well as instruct the inexperienced reader to be told that

Dr. Beddoes, who pointed out in strong terms the too great exclusiveness of Dr. Clutterbuck's theory, fell himself into the gastro-enteritic error, and with quite as little discrimination advocated depletory measures directed to the abdomen, particularly by leeches in "relays of dozens;" as it were foreshadowing the theory and the practice of the school of Broussais. Thus do medical reasoners for ever seem to be following one another in circles, intersected by other circles, tangent in error or in truth; like unto blindfolded men, propelling a load of theory, with strenuous efforts to preserve a straight line, but deviating to the right or left, or retrograde, as the inequality of the course determine.

---

